# Differences in pain perception, health-related quality of life, disability, mood, and sleep between Brazilian and Spanish people with chronic non-specific low back pain

**DOI:** 10.1590/bjpt-rbf.2014.0175

**Published:** 2016-06-16

**Authors:** Daiana P. Rodrigues-De-Souza, César Fernández-De-Las-Peñas, Francisco J. Martín-Vallejo, Juan F. Blanco-Blanco, Lourdes Moro-Gutiérrez, Francisco Alburquerque-Sendín

**Affiliations:** 1 Department of Physical Therapy, Universidad Católica de Ávila, Avila, Spain; 2 Department of Physical Therapy, Occupational Therapy, Physical Medicine and Rehabilitation, Universidad Rey Juan Carlos, Alcorcón, Spain; 3 Cátedra de Investigación y Docencia en Fisioterapia: Terapia Manual y Punción Seca, Universidad Rey Juan Carlos, Alcorcón, Spain; 4 Department of Statistics, Universidad de Salamanca, Salamanca, Spain; 5 Instituto de Investigación Biomédica de Salamanca (IBSAL), Salamanca, Spain; 6 Hospital Universitario de Salamanca Unidad Virgen Vega, Salamanca, Spain; 7 Department of Social Psychology and Anthropology, Universidad de Salamanca, Salamanca, Spain; 8 Department of Nursing and Physical Therapy, Universidad de Salamanca, Salamanca, Spain

**Keywords:** back pain, cultural characteristics, health evaluation, disabled persons, affect

## Abstract

**Background:**

Cultural and social factors play an important role in the development and persistence of Low Back Pain (LBP). Nevertheless, there are few studies investigating differences in LBP features between countries.

**Objective:**

To determine differences in pain perception between individuals with LBP living in Brazil and Spain.

**Method:**

Thirty Spanish individuals and 30 age- and sex-comparable Brazilian individuals with LBP were recruited from the Public Health Services of both countries. The Numerical Pain Rating Scale and the pain rating index (PRI), the number of words chosen (NWC), and the present pain index (PPI) extracted from the McGill Pain Questionnaire were used to assess pain. The Oswestry Disability Index, the Short Form-36, Beck Depression Inventory-II, and Pittsburgh Sleep Quality Index were also applied. Differences between countries and the correlation between demographic and clinical variables in each country were assessed with parametric and the nonparametric tests.

**Results:**

A significant Country by Gender interaction was found for the PRI total score (P=0.038), but not for intensity of pain, disability, PPI, or NWC, in which Spanish women exhibited greater pain ratio than Spanish men (P=0.014), and no gender differences were identified in Brazilians. The Spanish group showed a consistent pattern of correlations for clinical data. Within Brazilian patients, fewer correlations were found and all of the coefficients were lower than those in the Spanish group.

**Conclusion:**

The pain perception in patients with LBP is different depending on the country. Within Spanish patients, LBP is considered a more global entity affecting multidimensional contexts.

## BULLET POINTS

- Pain perception in chronic LBP could be different depending on the country.
- Spanish women present greater pain and poorer mental health than Spanish men do.
- Spanish patients consider that LBP affects multidimensional contexts.
- The potential relevance of cultural factors in chronic LBP must be determined.

## Introduction

Low back pain (LBP) refers to symptoms located under the costal edge and above the folds of the lower gluteus muscles with or without irradiation to the leg^1^. LBP is classified as chronic when it persists for over 12 weeks^2^. Non-specific LBP is not attributed to any recognizable pathology (e.g., infection, tumor, osteoporosis, rheumatoid arthritis, fracture, or inflammation) and it is much more frequent than specific LBP derived from underlying medical conditions^2^ ^,^ ^3^.

Pain is a complex perceptive disorder associated with social and cultural factors^4^ ^,^ ^5^. In fact, recognition of the pain experience is a complex subjective process with important psychosocial and cultural influences. In recent years, researchers have become highly interested in the effects of cultural factors^6^ ^,^ ^7^, since these factors have acquired greater importance in chronic pain due to the biopsychosocial model^8^ ^,^ ^9^.

Although no representative study provides an overall prevalence of LBP in Brazil^10^, some studies have determined a prevalence of LBP in this country ranging from 4.2%^11^ to 63%^12^. As an example, the prevalence of LBP in Pelotas, a medium-sized city of Brazil, has increased from 4.2% to 9.6% within the 2002-2010 period, in part due to an increase in life expectancy in this country^13^. Being a woman, smoker, and being married^13^ have been identified as risk factors for LBP, whereas age, body mass index (BMI), exposure to repetitive movements, working in uncomfortable postures^13^, and having sleep alterations are positively associated with LBP^14^. In Spain, the prevalence of LBP is around 20% and has remained unvaried in the last five-year period^15^. As in Brazil, LBP was associated with being a woman, increasing age, low educational level, low earning, and lack of physical activity^16^.

Although it seems that cultural, social, and geographic factors play an important role in the development and persistence of LBP^17^ and other musculoskeletal complaints^18^, there are few studies investigating differences in LBP between Europe and South America, probably due to the difficulty in objectively measuring cultural impact in pain^19^. The research in this area observed medical, physical, and psychosocial differences among patients with LBP^20^ and other musculoskeletal complaints^21^ ^-^ ^23^ in different cultures, societies, and countries. Nevertheless, there is a lack of studies addressing the multi-dimensional features of the LBP presentation in this area^24^.

Therefore, the main objective of the current study was to determine the differences in pain perception between individuals with LBP living in Brazil and Spain. The secondary objective was to determine the differences in health-related quality of life, disability, mood, and sleep quality between both countries and to identify the relations between demographic and clinical data in each country.

## Method

### Participants

A cross-sectional design was used in this study. Thirty patients with LBP recruited from the Orthopedic Service at the São Carlos Health Unit (Brazilian Public Health Service) and 30 age- and sex-comparable individuals with LBP recruited from the Orthopedic Hospital Department at University-Hospital of Salamanca (Spanish Public Health Service) were included. Participants were recruited through consecutive sampling. To be eligible, they had to suffer from non-specific LBP without referral into the lower extremity for more than 6 months and diagnosed by an orthopedic physician. Exclusion criteria included: 1) age less than 18 years; 2) LBP with a specific underlying medical pathology such as tumor, infection, inflammatory disorders, disc problems, and nerve root compromise; 3) diagnosis of fibromyalgia syndrome; 4) previous lumbar spine surgery; 5) diagnosis of psychiatric illness; 6) presence of other musculoskeletal diagnosis at the time of the study; or 7) refusal to participate in the study. The medical history from each patient was requested from their primary care physician to assess the presence of the exclusion criteria. The same interviewer (DPRS), who was bilingual in Brazilian and Spanish languages and had 6 years of clinical experience, conducted the data collection in both countries. All of the questionnaires were self-administered.

All participants read and signed an informed consent form before their inclusion in the study. The current study was conducted following the Declaration of Helsinki and was approved by the Ethics Committee of the University of Salamanca, Salamanca, Spain (code number: 7/2-12). In addition, clinical services of both countries accepted these ethical considerations following an international ethical agreement between both countries.

### Pain outcomes

An 11-point numerical pain rating scale (NPRS, 0: no pain; 10: maximum pain) was used to assess the mean intensity of pain experienced in the last week^25^. Differences below 1.5 points in the NPRS are not considered clinically relevant^26^. Patients were also asked about the duration of pain symptoms.

The McGill Pain Questionnaire (MPQ) was used to assess pain intensity. Items were joined to achieve three main domains of the questionnaire, i.e., sensory (1-15), affective (16-18), and evaluative (19), as well as the total score or pain rating index (PRI) for each domain. We also considered the number of words chosen by the patient (NWC) and the present pain index (PPI), which describes current pain intensity from 0 (no pain) to 5 (excruciating pain) scales. In the current study, we used the Spanish^27^ and the Brazilian^28^ versions of the questionnaire, which have shown good validity. A 30% difference is generally considered clinically meaningful^29^.

### Oswestry Disability Index (ODI)

The ODI is one of the most frequently used tools for measuring LBP-related disability^30^ ^,^ ^31^. The ODI contains 10 questions (rated from 0 to 5 points each) about daily activities, including inventories of pain intensity, personal care, lifting, walking, sitting, standing, sleeping, sexual life, social life, and traveling. The ODI scores range from 0 to 100. Higher scores indicate greater disability. The ODI was validated for LBP and it presents high reliability and consistency (ICC=0.99, α=0.87)^32^. In the current study, we also used the Brazilian^33^ ^,^ ^34^ and Spanish^35^ adapted versions of this questionnaire. It has been determined that a minimal clinically important difference (MCID) of the ODI is a difference of 10 points and more than 30%^29^.

### Health-related quality of life

The health-related quality of life was assessed with the Medical Outcomes Study Short Form 36 (SF-36) questionnaire, which assessed eight domains: physical functioning, role physical, bodily pain, general health, vitality, social functioning, role-emotional, and mental health^36^. After adding the Likert-scaled items, each scale is standardized, so that it ranges from 0 (lowest level of functioning) to 100 (highest level)^37^. Again, the Spanish^38^ and Brazilian^39^ adaptations were used.

The SF-36 questionnaire can be also summarized as two health measures: physical component score (PCS) and mental component score (MCS). The PCS is derived from the following domains (physical function, physical role, bodily pain, and general health), whereas the MCS is derived from emotional role, social function, mental health, and vitality domains^40^. In the current study, we considered physical and mental components of the SF36 questionnaire for the main analysis. As recommended by the IMMPACT group, a 30% change could be used as a barometer of positive clinical difference^41^.

### Beck Depression Inventory-II (BDI-II)

Participants also completed the Beck Depression Inventory-II (BDI-II), a 21-item self-report questionnaire that assesses affective, cognitive, and somatic symptoms of depression^42^. The BDI-II showed good internal consistency (α=0.83) and test-retest reliability (0.68-0.89)^42^. The Spanish^43^ and Brazilian^44^ versions have also shown good internal consistency. The MCID of the BDI-II is 17.5%^45^.

### Sleep quality assessment

The Pittsburgh Sleep Quality Index (PSQI) is the most commonly used tool for a comprehensive assessment of sleep quality^46^. The PSQI appraises sleep quality over a one-month period through a questionnaire differentiating between good and poor sleepers. It includes 19 self-rated questions and 5 questions answered by bedmates/roommates. The items use varying response categories that include recording usual bed time, usual wake time, number of actual hours slept, and number of minutes to fall asleep, as well as forced-choice Likert-type responses (0-3). The sum of the scores for the components yields one global score, which ranges from 0 to 21, where higher scores indicate worse sleep quality^47^. A total score >8.0 indicates poor sleep quality^47^. Buysse et al. ^47^ reported that the PSQI has good internal consistency (α=0.83) and test-retest reliability (r=0.85). The Brazilian^48^ and Spanish^49^ versions were used. No MCID data available for the PSQI.

### Sample size calculation

The sample size determination was performed using the Spanish software Ene 3.0 (Glaxo Smith Kline, Universidad Autónoma, Barcelona, Spain). The calculations were based on detecting clinical differences of 10 points in the ODI and standard deviation of 13 points^29^ between groups, with a bilateral significance level of 0.05 and power of 0.80. This generated a sample size of at least 28 patients in each group.

### Statistical analysis

Data were analyzed with the statistical package SPSS version 21.0 (SPSS Inc., Chicago, IL, USA). Descriptive data were collected for all patients. The Kolmogorov-Smirnov test was used to analyze the normal distribution of the variables. Quantitative data without a normal distribution (BDI and MCS) were analyzed with nonparametric tests, and the remaining data with normal distribution were analyzed with parametric tests. Differences in the quantitative variables between countries were assessed with the unpaired Student-t test and the nonparametric Mann-Whitney U-test. The chi-square (χ2) test was used to analyze differences in sex between both groups. A 2-way analysis of variance (ANOVA) test was used to investigate the differences in outcomes with country (Brazil or Spain) and gender (men or women) as the between-subjects factors. The Pearson (r) test and Spearman’s rho (rs) test were used to determine the correlations between demographic and clinical variables in each country. The strength of the correlations was classified as weak (r from 0.1 to 0.3), moderate (r from 0.4 to 0.6), or strong (r from 0.7 to 1)^50^. The statistical analysis was conducted at 95% confidence level.

## Results

### Differences in demographic and clinical data by country and by gender

No significant differences between patients of either country were observed for age (P=0.64), gender distribution (P=0.80, weight (P=0.14), height (P=0.06), BMI (P=0.85), years with pain (P=0.74), pain intensity (P=0.24), PPI (P=0.92), PRI total score (P=0.72), or NWC (P=0.11). On the other hand, we found greater disability in Spanish patients than in Brazilian people (mean difference MD, 95%CI: 9.87, 18.06-1.68), P=0.02). Table 1 summarizes demographic and clinical data of Spanish and Brazilian patients with LBP.

**Table 1:** Demographic and clinical data of Brazilian and Spanish patients with low back pain (LBP).

	**Brazilian (n=30)**	**Spanish (n=30)**	**Significance**
**Gender (male/female)**	13 / 17	14 / 16	X^2^=0.07; P=0.795
**Age (years)**	49.7±10.5 (45.8-53.6)	51.1±13 (46.3-56)	t=-0.469; P=0.641
**Weight (kg)**	74±7.5 (71.2-76-8)	76.8±6.5 (74.4-79.2)	t=-1.515; P=0.135
**Height (cm)**	165.4±0.1 (162.8-167.9)	169±0.1 (166.3-171.7)	t=-2.003; P=0.06
**BMI (kg/cm^2^)**	27.1±2.6 (26.1-28.1)	27.0±2.8 (25.9-28)	t=0.188; P=0.852
**Time suffering pain (months)**	117.6±94.7 (82.2-152.9)	125.9±102.6 (87.6-164.2)	t=-0.328; P=0.744
**Current pain intensity (NPRS, 0-10)**	7.1±1.6 (6.5-7.7)	6.4±2.5 (5.5-7.4)	t=1.185; P=0.241
**PPI (MPQ, 0-5)**	3.1±1.1 (2.7-3.5)	3.1±1.4 (2.6-3.6)	t=0.1; P=0.921
**PRI total score (MPQ, 0-100)**	41.3±15.9 (35.4-47.3)	43.2±23.6 (34.3-52)	t=-0.357; P=0.723
**NWC (MPQ, 0-20)**	14.2±3.9 (12.8-15.7)	12.3±5.1 (10.4-14.2)	t=1.641; P=0.107
**PCS (SF-36, 0-100)** ^*^	59.6±20.7 (51.9-67.3)	42.5±22.2 (34.3-50.8)	t=3.081; P=0.001
**MCS (SF-36, 0-100)**	70.5±16.7 (64.2-76.7)	62.6±21.1 (54.8-70.5)	z=-1.02; P=0.308
**ODI score (0-100)** ^*^	24.4±13.3 (19.4-29.4)	34.3±18 (27.5-41)	t=-2.411; P=0.019
**BDI score (0-63)**	9.5±5.8 (7.4-11.6)	11.1±9.4 (7.6-14.6)	z=-0.03; P=0.976
**PSQI score (0-21)**	8±2.3 (7.1-8.9)	7.9±4.4 (6.3-9.6)	t=0.073; P=0.942

Data are expressed as means ± standard deviation (95% Confidence Interval); BMI: Body Mass Index; PPI: Present Pain Index; NWC: Number of Words Chosen; PRI: Pain Rating Index; PCS: Physical Component Summary; MCS: Mental Component Summary; ODI: Oswestry Disability Index; BDI: Beck Depression Inventory; PSQI: Pittsburgh Sleep Quality Index.

* Indicates statistically significant difference between groups.

The ANOVA revealed a significant Country * Gender interaction for PRI total score (P=0.04), but not for intensity of pain (P=0.88), disability (P=0.35), PPI (P=0.75), or NWC (P=0.06). Spanish women with LBP exhibited greater pain ratio than Spanish men (MD, 95%CI: 18.06, 3.84-32.28, P=0.01), whereas no gender differences were found in Brazilians. Table 2 shows demographic and clinical data of Spanish and Brazilian patients with LBP by gender.

**Table 2:** Clinical data of Brazilian and Spanish patients with low back pain (LBP) by gender.

	**Brazilian**		**Spanish**	
	**Women (n=17)**	**Men (n=13)**	**Women (n=16)**	**Men (n=14)**
**Current pain (NPRS, 0-10)**	7.5±1.7 (6.5-8.5)	6.6±1.3 (5.4-7.7)	6.9±2.4 (5.9-8.0)	5.9±2.6 (4.7-7.0)
**PPI (MPQ, 0-5)**	3.2±1.1 (2.7-3.8)	3.0±1.2 (2.3-3.7)	3.3±1.5 (2.5-4.1)	2.9±1.4 (2.1-3.6)
**PRI total score (MPQ, 0-100)** ^*^	39.9±21.1 (30.5-49.3)	43.2±18.2 (32.4-54.0)	51.6±22.1 (41.9-61.3)	33.6±22.4 (23.2-43.9)
**NWC (MPQ, 0-20)**	14.2±4.3 (12.0-16.4)	14.2±3.6 (12.1-16.4)	14.3±4.7 (11.8-16.8)	10.0±4.7 (7.3-12.7)
**PCS (SF-36, 0-100)**	62.1±20.7 (51.7-72.5)	56.4±21.1 (44.5-68.3)	37.8±20.0 (27.1-48.5)	48.0±24.0 (36.5-59.4)
**MCS (SF-36, 0-100)** ^*^	73.0±17.2 (64.2-81.9)	67.1±16.1 (56.9-77.2)	54.8±21.5 (45.6-63.9)	71.6±17.2 (61.9-81.4)
**ODI score (0-100)**	24.2±15.2 (16.5-31.9)	24.7±10.9 (15.9-33.5)	37.7±14.6 (29.7-45.7)	30.4±21.1 (21.8-38.9)
**BDI score (0-63)** ^*^	8.5±6.7 (4.9-12.0)	10.9±4.1 (6.8-14.9)	14.9±9.6 (11.3-18.6)	6.6±7.1 (2.8-10.5)
**PSQI score (0-21)** ^*^	7.9±2.6 (6.3-9.6)	8.1±1.9 (6.2-10.0)	9.5±4.2 (7.8-11.2)	6.1±4.1 (4.3-8.0)

Data are expressed as means ± standard deviation (95% Confidence Interval); BMI: Body Mass Index; PPI: Present Pain Index; NWC: Number of Words Chosen; PRI: Pain Rating Index; PCS: Physical Component Summary; MCS: Mental Component Summary; ODI: Oswestry Disability Index; BDI: Beck Depression Inventory; PSQI: Pittsburgh Sleep Quality Index.

* Indicated statistically significant group by gender interaction.

### Quality of life

Significant differences between countries were observed for physical (MD, 95%CI: 17.06, 5.98-28.15, P<0.01) but not for mental (P=0.31) component scores of the SF36 questionnaire: Spanish patients exhibited lower physical quality of life than Brazilian patients (Table 1). When gender was included, the ANOVA found a significant Country * Gender interaction for the MCS (P=0.02), but not for the PCS (P=0.16). Spanish women exhibited lower MCS than Spanish men (MD, 95%CI: 16.87, 3.49-30.24) and Brazilian men (MD, 95%CI: 12.31, 0.46-25.08) and women (MD, 95%CI: 18.26, 5.53-30.99) (Table 2).

### Depression and sleep quality

No significant differences between countries were observed for depressive symptoms (P=0.98) or sleep quality (P=0.94) (Table 1). When gender was included, the ANOVA revealed significant Country * Gender interactions for both depression (P=0.01) and sleep quality (P=0.04). Again, Spanish women exhibited higher levels of depression and worse sleep quality than Spanish men did (BDI: MD, 95%CI: 8.30, 2.96-13.63; PSQI: 3.36, 0.87-5.85) and Brazilian women (BDI: MD, 95%CI: 6.47, 1.39-11.55; PSQI: 1.6, 0.11-3.13) (Table 2).

### Correlations between demographic and clinical data by country

Significant positive correlations were found between age and BMI, ODI, NPRS, PRI, and PPI in Brazilian, but not Spanish, patients with LBP. Within Brazilian patients, the older the patients were, the greater the BMI, disability, and pain intensity. Age was negatively associated with PCS in Brazilian, but not Spanish, patients. The older the patients were, the lower the physical component of quality of life (Tables 3 and 4).

**Table 3:** Correlations between demographic and clinical data within Spanish patients with low back pain (LBP).

**(n=30)**	**Age**	**BMI**	**PSQI**	**ODI**	**NPRS**	**PRI**	**NWC**	**PPI**	**BDI**	**PCS**
**BMI**	0.267									
**PSQI**	0.023	0.308								
**ODI**	0.215	0.016	0.461^*^							
**NPRS**	–0.201	–0.387^*^	0.364^*^	0.427^*^						
**PRI**	–0.205	–0.274	0.486^**^	0.621^**^	0.743^**^					
**NWC**	–0.233	–0.174	0.553^**^	0.583^**^	0.600^**^	0.919^**^				
**PPI**	0.005	–0.158	0.349	0.374^*^	0.592^**^	0.599^**^	0.480^**^			
**BDI**	0.271	–0.056	0.458^*^	0.597^**^	0.324	0.622^**^	0.694^**^	0.179		
**PCS**	–0.306	0.006	–0.313	–0.611^**^	–0.259	–0.426^*^	–0.421*	–0.159	–0.664^**^	
**MCS**	–0.252	–0.179	–0.508^**^	–0.574^**^	–0.372^*^	–0.576^**^	–0.664^**^	–0.285	–0.752^**^	0.767^**^

Values are expressed as Pearson’s *r* or Spearman’s rho; NS: not significant.

* P<0.05.

** P<0.01.

**Table 4:** Correlations between demographic and clinical data within Brazilian patients with low back pain (LBP).

**(n=30)**	**Age**	**BMI**	**PSQI**	**ODI**	**NPRS**	**PRI**	**NWC**	**PPI**	**BDI**	**PCS**
**BMI**	0.490^**^									
**PSQI**	0.153	0.011								
**ODI**	0.681^**^	0.364^*^	0.123							
**NPRS**	0.428^*^	0.196	0.311	0.587^**^						
**PRI**	0.437^*^	0.098	0.268	0.473^**^	0.328					
**NWC**	0.25	0.057	0.072	0.448^*^	0.307	0.766^**^				
**PPI**	0.435^*^	0.093	0.161	0.522^**^	0.336	0.297	0.191			
**BDI**	0.216	0.086	0.138	0.399^*^	0.188	0.354	0.431^*^	0.099		
**PCS**	–0.567^**^	–0.181	–0.354	–0.643^**^	-0.304	–0.428^*^	–0.27	–0.615^**^	–0.393^*^	
**MCS**	–0.132	–0.100	–0.385^*^	–0.248	0.050	–0.203	–0.191	–0.202	–0.504^**^	0.655^**^

Values are expressed as Pearson’s *r* or Spearman’s rho; NS: not significant.

* P<0.05.

** P<0.01.

The Spanish group showed a consistent pattern of correlations for clinical data, where all variables were significantly related to each other, with a few exceptions including PSQI to PCS, NPRS to BDI and PCS, and PPI to PSQI. All correlation coefficients were considered moderate or high (Table 3).

In contrast, within Brazilian patients, the correlation pattern depended on the outcome. Thus, the ODI was the only variable related to all of the others, with the exception of the MCS: the greater the disability was, the worse the pain intensity, sleep, physical quality of life, and mood. The PCS also showed negative correlations with PRI, PPI, BDI, and MCS: the worse the physical aspect of quality of life, the worse pain intensity, mood, or sleep quality. In general, correlations within Brazilian patients were lower than those in the Spanish group (Table 4).

## Discussion

The results of the current study show that pain perception in patients with LBP can be affected in some aspects depending on the country of residence. We observed that the country of origin and gender do not determine a different pattern of behavior for physical quality of life and disability; however, the mental quality of life and mood of Spanish women suffering from LBP was appreciably worse than those of Brazilian men and women and Spanish men. Further, the correlation patterns seen in Spanish patients suggest that LBP could be a global phenomenon affecting several components of the subject’s life, whereas this correlation is less clear for Brazilians, at least for the studied sample.

### Between-countries differences

Pain intensity did not show significant differences between the two countries. In fact, differences in the NPRS did not achieve the threshold to be considered clinically relevant^26^. In contrast, Spanish individuals exhibited statistically significant and clinically higher levels of disability than Brazilians, with differences above the MCID^29^. This higher self-rated perception of disability in Spain may be associated with sedentary lifestyle^15^ or a social security system associated with job absenteeism^51^. This assumption is supported in other northern European countries where these factors have been associated with development and persistence of LBP^7^.

Our sample of Spanish patients reported a similar physical health-related quality of life status than in other countries^52^; nonetheless, the physical, but not mental, quality of life of Spanish patients was worse than that of Brazilians.

No differences in depressive levels were observed between Brazilian and Spanish patients with LBP. The prevalence of depression in patients with LBP is high^53^, suggesting that this psychological state may involve common mechanisms with chronic pain^54^. Nevertheless, we should recognize that our patients with LBP exhibited lower levels of depression (9<BDI-II<11). We do not know if the presence of higher depressive levels would show between-countries differences.

In addition, Brazilian and Spanish patients with LBP exhibited similar patterns of sleep quality. In our study, most of the patients showed around 8 points suggesting that both populations exhibited poor sleep quality^55^. Our results agree with previous studies supporting that people with LBP have poor sleep quality and high levels of insomnia^56^ ^,^ ^57^. The relationship between pain and sleep disorders is bidirectional since sleep disturbances may increase pain, but pain may also cause sleep disorders^58^. Thus, the lack of sleep may interfere with pain processing^59^.

### Gender

It is well accepted that gender is a relevant factor in the modulation of pain, and there is a considerable body of evidence suggesting that women have more frequent LBP^60^ ^-^ ^62^, higher levels of disability^63^, a and higher number of comorbidities^64^ than men.

In the current study, gender was a differentiating factor between countries in some outcomes. For instance, although the intensity of LBP and the NWS did not reveal differences by gender and country, Spanish women reported higher PRI^29^ ^,^ ^65^ than Spanish men whereas Brazilian women and men had similar values. There is evidence that culture/race interacts with gender in the pain experience^66^ ^,^ ^67^ and this influence is modulated by the country in which the subject grows up. It has been also described that perceived disability may differ according to gender^68^ ^,^ ^69^ since women are more likely to experience disability than men^63^. However, we did not observe differences in self-perceived disability between men and women in either country. Nevertheless, more research is needed to determine why chronic pain is more likely to be disabling for women than for men^54^.

We observed that Spanish women reported worse mental, but not physical, health status than Brazilian women and Spanish men. These data are partly in agreement with those of other authors, in which Italian women also reported poorer mental and physical health^52^. In this study, Italian men separated pain from purely physical deterioration, showing that LBP has important mental health repercussions. Again, it is reported that women have more co-morbid mood disorders^70^, more co-morbid physical conditions^71^, and a higher number of somatic symptoms^72^ than men do. In our study, this profile may have only applied to Spanish women, who displayed the worst depressive state and greatest sleep disorders than the rest of the subjects.

Different theories are proposed to explain gender differences in pain perception. First, biological factors may interfere in the greater frequency of chronic pain in women^73^ ^,^ ^74^. Second, regarding interference in social functions, women may perceive the painful event as a threat owing to the multiple roles and responsibilities they are charged with, such as child and elderly care, household management, and job^62^, even though women are considered more resistant to expressing their pain^60^ and more emotional than men^75^. By contrast, men may downplay their own pain, highlighting that insensitivity or resistance to pain is a sign of masculine virility^60^ ^,^ ^69^. These hypotheses clearly reflect the cultural influence on the experience of pain.

### Self-perception of the pain condition

We tried to determine the potential global self-perception of the condition by investigating the correlation between different outcomes. We found several correlations within Spanish patients with LBP that were less significant in Brazilian people. These findings indicate that LBP seems to be considered a global condition by Spanish people with several domains affected to the same extent, as previously described^76^. The relationships observed within Spanish patients agree with previous findings, i.e., patients’ quality of life more dependent on the degree of disability they self-perceived than the actual pain intensity^77^ ^,^ ^78^. Therefore, the study of a multidimensional set of factors, i.e., quality of life, mood, and sleep, would be more important than the study of pain itself.

According to our results, when dealing with LBP it seems necessary to include the social and cultural aspects in the clinical context since this may offer an improvement in the therapeutic approach and the long-term clinical results.

### Limitations

We should recognize some potential limitations in the current study. First, the sample size can be considered small, which could decrease the power of multiple comparisons; however, our main objective was not to conduct a population-based study. Future studies including more representative populations are now needed to determine the influence of cultural factors on LBP. Secondly, the clinical heterogeneity of the patients could also influence the results. Further, social variables were neither collected nor studied, which limits the applicability of the results. Third, we did not collect outcomes on catastrophism or kinesiophobia, two clinical topics relevant to patients with LBP. Finally, the questionnaires used in the study were not specific for LBP patients, with the exception of the ODI. Other questionnaires could afford new perspectives on the results.

## Conclusions

This study showed that pain perception in patients with LBP is affected in some aspects depending on the country of residence. We found that Spanish women reported greater pain index, poorer mental health, poorer mood, and a poorer quality of life than Spanish men and Brazilian patients. No differences in pain intensity and disability were observed. The level of relationships between pain, disability, quality of life, mood, and sleep patterns was significantly higher in Spanish patients with LBP, suggesting that this condition is considered a global entity affecting multidimensional contexts within Spanish patients. Future population-based studies are needed to determine the potential relevance of cultural factors in patients with LBP.

## References

[1] Anderson JA (1977). Problems of classification of low-back pain. Rheumatol Rehabil.

[2] Chou R (2011). Low back pain (chronic). Am Fam Physician.

[3] Seguí M, Gérvas J (2002). El dolor lumbar. Semergen.

[4] Campbell CM, Edwards RR (2012). Ethnic differences in pain and pain management. Pain Manag..

[5] Lasch KE (2002). Culture and pain. Pain Clinical Updates..

[6] Edwards CL, Fillingim RB, Keefe F (2001). Race, ethnicity and pain. Pain.

[7] Vargas-Prada S, Serra C, Martínez JM, Ntani G, Delclos GL, Palmer KT (2013). Psychological and culturally-influenced risk factors for the incidence and persistence of low back pain and associated disability in Spanish workers: findings from the CUPID study. Occup Environ Med.

[8] Turk DC, Turk DC (1996). Biopsychosocial perspective on chronic pain. Psychological approaches to pain management: a practitioner’s handbook.

[9] Turk DC, Okifuji A (2002). Psychological factors in chronic pain: evolution and revolution. J Consult Clin Psychol.

[10] Nascimento PR, Costa LO (2015). Low back pain prevalence in Brazil: a systematic review. Cad Saude Publica.

[11] Silva MC, Fassa AG, Valle NC (2004). Chronic low back pain in a Southern Brazilian adult population: prevalence and associated factors. Cad Saude Publica.

[12] Ferreira GD, Silva MC, Rombaldi AJ, Wrege ED, Siqueira FV, Hallal PC (2011). Prevalence and associated factors of back pain in adults from southern Brazil: a population-based study. Rev Bras Fisioter..

[13] Meucci RD, Fassa AG, Paniz VM, Silva MC, Wegman DH (2013). Increase of chronic low back pain prevalence in a medium-sized city of southern Brazil. BMC Musculoskelet Disord.

[14] Zanuto EA, Codogno JS, Christófaro DG, Vanderlei LC, Cardoso JR, Fernandes RA (2015). Prevalence of low back pain and associated factors in adults from a middle-size Brazilian city. Cien Saude Colet.

[15] Fernández-de-Las-Peñas C, Alonso-Blanco C, Hernández-Barrera V, Palacios-Ceña D, Jiménez-García R, Carrasco-Garrido P (2013). Has the prevalence of neck pain and low back pain changed over the last 5 years? A population-based national study in Spain. Spine J.

[16] Fernández-de-las-Peñas C, Hernández-Barrera V, Alonso-Blanco C, Palacios-Ceña D, Carrasco-Garrido P, Jiménez-Sánchez S (2011). Prevalence of neck and low back pain in community-dwelling adults in Spain: a population-based national study. Spine (Phila Pa 1976)..

[17] Aceves-Avila FJ, Ferrari R, Ramos-Remus C (2004). New insights into culture driven disorders. Best Pract Clin Res Rheumatol..

[18] Farioli A, Mattioli S, Quaglieri A, Curti S, Violante FS, Coggon D (2014). Musculoskeletal pain in Europe: the role of personal, occupational, and social risk factors. Scand J Work Environ Health.

[19] Free MM (2002). Cross-cultural conceptions of pain and pain control. BUMC Proc.

[20] Sanders SH, Brena SF, Spier CJ, Beltrutti D, McConnell H, Quintero O (1992). Chronic low back pain patients around the world: cross-cultural similarities and differences. Clin J Pain.

[21] Carugno M, Pesatori AC, Ferrario MM, Ferrari AL, Silva FJ, Martins AC (2012). Physical and psychosocial risk factors for musculoskeletal disorders in Brazilian and Italian nurses. Cad Saude Publica.

[22] Coggon D, Ntani G, Palmer KT, Felli VE, Harari R, Barrero LH (2013). Disabling musculoskeletal pain in working populations: is it the job, the person, or the culture?. Pain.

[23] Straube S, Croft P (2013). Musculoskeletal pain in different occupational groups and different countries. Pain.

[24] Billis EV, McCarthy CJ, Oldham JA (2007). Subclassification of low back pain: a cross-country comparison. Eur Spine J.

[25] Jensen MP, Turner JA, Romano JM, Fisher LD (1999). Comparative reliability and validity of chronic pain intensity measures. Pain.

[26] Kovacs FM, Abraira V, Royuela A, Corcoll J, Alegre L, Cano A (2007). Minimal clinically important change for pain intensity and disability in patients with nonspecific low back pain. Spine (Phila Pa 1976)..

[27] Lázaro C, Caseras X, Whizar-Lugo VM, Wenk R, Baldioceda F, Bernal R (2001). Psychometric properties of a Spanish version of the McGill Pain Questionnaire in several Spanish-speaking countries. Clin J Pain.

[28] Costa LCM, Maher CG, McAuley JH, Hancock MJ, Oliveira WM, Azevedo DC (2011). The Brazilian-Portuguese versions of the McGill Pain Questionnaire were reproducible, valid, and responsive in patients with musculoskeletal pain. J Clin Epidemiol.

[29] Ostelo RW, Deyo RA, Stratford P, Waddell G, Croft P, Von Korff M (2008). Interpreting change scores for pain and functional status in low back pain: towards international consensus regarding minimal important change. Spine (Phila Pa 1976)..

[30] Macdowell I, Newell C (1996). Measuring health: a guide to rating scales and questionnaires.

[31] Roland M, Morris R (1983). A study of the natural history of back pain. Part I: development of a reliable and sensitive measure of disability in low-back pain. Spine (Phila Pa 1976).

[32] Roland M, Fairbank J (2000). The Roland-Morris Disability Questionnaire and the Oswestry Disability Questionnaire. Spine (Phila Pa 1976).

[33] Coelho RA, Siqueira FB, Ferreira PH, Ferreira ML (2008). Responsiveness of the Brazilian-Portuguese version of the Oswestry Disability Index in subjects with low back pain. Eur Spine J.

[34] Vigatto R, Alexandre NM, Correa HR (2007). Development of a Brazilian Portuguese version of the Oswestry Disability Index: cross-cultural adaptation, reliability, and validity. Spine (Phila Pa 1976).

[35] Borrego PS, Sáez ML, Borrego JM, Borrego PA, Borrego P (2005). Psychometric analysis of the Oswestry lower back pain disability questionnaire. Fisioterapia..

[36] Ware JEJ, Sherbourne CD (1992). The MOS 36-item short-form health survey (SF-36). I. Conceptual framework and item selection. Med Care.

[37] Ware JEJ, Snow KK, Kosinski M, Gandek B (1993). SF-36 Health Survey: manual and interpretation guide.

[38] Alonso J, Regidor E, Barrio G, Prieto L, Rodríguez C, Fuente L (1998). Population reference values of the Spanish version of the Health Questionnaire SF-36. Med Clin.

[39] Mesquita R, Bosi M, Santos W, Meinão I, Rodrigues M (1999). Tradução para língua protuguesa e validação do questionário genérico de avalição de qualidade de vida SF-36 (Brasil SF-36). Rev Bras Reumatol.

[40] Ware JE, Kosinski M, Keller S (1994). SF-36 physical and mental summary scales: a user's manual.

[41] Dworkin RH, Turk DC, Farrar JT, Haythornthwaite JA, Jensen MP, Katz NP (2005). Core outcome measures for chronic pain clinical trials: IMMPACT recommendations. Pain.

[42] Beck AT, Steer RA, Brown GK (1996). Beck depression inventory.

[43] Azocar F, Areán P, Miranda J, Muñoz RF (2001). Differential item functioning in a Spanish translation of the Beck Depression Inventory. J Clin Psychol.

[44] Gomes-Oliveira MH, Gorenstein C, Lotufo F, Andrade LH, Wang YP (2012). Validation of the Brazilian Portuguese version of the Beck Depression Inventory-II in a community sample. Rev Bras Psiquiatr.

[45] Button KS, Kounali D, Thomas L, Wiles NJ, Peters TJ, Welton NJ (2015). Minimal clinically important difference on the Beck Depression Inventory - II according to the patient’s perspective. Psychol Med.

[46] Cole JC, Dubois D, Kosinski M (2007). Use of patient-reported sleep measures in clinical trials of pain treatment: a literature review and synthesis of current sleep measures and a conceptual model of sleep disturbance in pain. Clin Ther.

[47] Buysse DJ, Reynolds CF, Monk TH, Berman SR, Kupfer DJ (1989). The Pittsburg Sleep Quality Index: a new instrument for psychiatric practice and research. Psychiatry Res.

[48] Bertolazi AN, Fagondes SC, Hoff LS, Dartora EG, Miozzo IC, Barba ME (2011). Validation of the Brazilian Portuguese version of the Pittsburgh Sleep Quality Index. Sleep Med.

[49] Jiménez-Genchi A, Monteverde-Maldonado E, Nenclares-Portocarrero A, Esquivel-Adame G, de la Vega-Pacheco A (2008). Confiabilidad y análisis factorial de la versión en español del índice de calidad de sueño de Pittsburgh en pacientes psiquiátricos. Gac Med Mex.

[50] Cohen J (1988). Statistical power analysis for the behavioral sciences.

[51] Salvans MM, González-Viejo MA (2008). Disability by low back pain in Spain from 2000 to 2004. Med Clin.

[52] Salaffi F, De Angelis R, Stancati A, Grassi W (2005). Health-related quality of life in multiple musculoskeletal conditions: a cross-sectional population based epidemiological study. II. The MAPPING study. Clin Exp Rheumatol.

[53] Sullivan MJ, Reesor K, Mikail S, Fisher R (1992). The treatment of depression in chronic low back pain: review and recommendations. Pain.

[54] Greenspan JD, Craft RM, LeResche L, Arendt-Nielsen L, Berkley KJ, Fillingim RB (2007). Studying sex and gender differences in pain and analgesia: a consensus report. Pain.

[55] Alsaadi SM, McAuley JH, Hush JM, Bartlett DJ, Henschke N, Grunstein RR (2013). Detecting insomnia in patients with low back pain: accuracy of four self-report sleep measures. BMC Musculoskelet Disord.

[56] O’Donoghue GM, Fox N, Heneghan C, Hurley DA (2009). Objective and subjective assessment of sleep in chronic low back pain patients compared with healthy age and gender matched controls: a pilot study. BMC Musculoskelet Disord.

[57] van de Water AT, Eadie J, Hurley DA (2011). Investigation of sleep disturbance in chronic low back pain: an age- and gender-matched case-control study over a 7-night period. Man Ther.

[58] Smith MT, Haythornthwaite JA (2004). How do sleep disturbance and chronic pain inter-relate? Insights from the longitudinal and cognitive-behavioral clinical trials literature. Sleep Med Rev.

[59] Lautenbacher S, Kundermann B, Krieg JC (2006). Sleep deprivation and pain perception. Sleep Med Rev.

[60] Budó ML, Nicolini D, Resta DG, Büttenbender E, Pippi MC, Ressel LB (2007). Culture permeating the feelings and the reactions in the face of pain. Rev Esc Enferm USP.

[61] Humbría A, Carmona L, Peña JL, Ortiz AM (2002). Impacto poblacional del dolor lumbar en España: resultados del estudio EPISER. Rev Esp Reumatol.

[62] Kreling MCGD, Cruz DALM, Pimenta CAM (2006). Prevalence of chronic pain in adult workers. Rev Bras Enferm.

[63] Von Korff M, Ormel J, Keefe FJ, Dworkin SF (1992). Grading the severity of chronic pain. Pain.

[64] LeResche L, Fillingim RB (2000). Epidemiologic perspectives on sex differences in pain. Sex, gender, and pain, progress in pain research and management.

[65] Farrar JT, Young JPJ, LaMoreaux L, Werth JL, Poole RM (2001). Clinical importance of changes in chronic pain intensity measured on an 11-point numerical pain rating scale. Pain.

[66] Fillingim RB (2005). Individual differences in pain responses. Curr Rheumatol Rep.

[67] Myers CD, Riley JL, Robinson ME (2003). Psychosocial contributions to sex-correlated differences in pain. Clin J Pain.

[68] Keogh E, McCracken LM, Eccleston C (2006). Gender moderates the correlation between depression and disability in chronic pain patients. Eur J Pain.

[69] Unruh AM (1996). Gender variations in clinical pain experience. Pain.

[70] Sloan DM, Kornstein SG (2003). Gender differences in depression and response to antidepressant treatment. Psychiatr Clin North Am.

[71] Paykel ES, Brugha T, Fryers T (2005). Size and burden of depressive disorders in Europe. Eur Neuropsychopharmacol.

[72] Von Korff M, Dworkin SF, Le Resche L, Kruger A (1988). An epidemiologic comparison of pain complaints. Pain.

[73] Berkley KJ, Zalcman SS, Simon VR (2006). Sex and gender differences in pain and inflammation: a rapidly maturing field. Am J Physiol Regul Integr Comp Physiol.

[74] Fillingim RB (2000). Sex, gender, and pain: women and men really are different. Curr Rev Pain.

[75] Helman C (2003). Cultura, saúde e doença.

[76] Ceran F, Ozcan A (2006). The relationship of the Functional Rating Index with disability, pain, and quality of life in patients with low back pain. Med Sci Monit.

[77] Kovacs FM, Abraira V, Zamora J, Fernández C (2005). The transition from acute to subacute and chronic low back pain: a study based on determinants of quality of life and prediction of chronic disability. Spine (Phila Pa 1976)..

[78] Kovacs FM, Abraira V, Zamora J, Teresa Gil del Real M, Llobera J, Fernández C (2004). Correlation between pain, disability, and quality of life in patients with common low back pain. Spine.

